# Therapeutic targeting in pediatric acute myeloid leukemia with aberrant *HOX*/*MEIS1* expression

**DOI:** 10.1016/j.ejmg.2023.104869

**Published:** 2023-10-29

**Authors:** Kristian L. Juul-Dam, Neerav N. Shukla, Todd M. Cooper, Branko Cuglievan, Olaf Heidenreich, E Anders Kolb, Milad Rasouli, Henrik Hasle, C Michel Zwaan

**Affiliations:** a Department of Pediatrics and Adolescent Medicine, Aarhus University Hospital, Aarhus, Denmark; b Department of Pediatrics, Memorial Sloan Kettering Cancer Center, New York, NY, USA; c Division of Hematology/Oncology, Seattle Children’s Hospital, University of Washington, Seattle, WA, USA; d Division of Pediatrics, The University of Texas MD Anderson Cancer Center, Houston, TX, USA; e Princess Máxima Center for Pediatric Oncology, Utrecht, the Netherlands; f Wolfson Childhood Cancer Research Centre, Translational and Clinical Research Institute, Newcastle University, Newcastle upon Tyne, UK; g Division of Oncology, Nemours/Alfred I. Dupont Hospital for Children, Wilmington, DE, USA; h Department of Pediatric Oncology, Erasmus MC-Sophia Children’s Hospital, Rotterdam, the Netherlands

**Keywords:** Acute myeloid leukemia, Children, *HOX*/*MEIS1* expression, Menin, Precision medicine

## Abstract

Despite advances in the clinical management of childhood acute myeloid leukemia (AML) during the last decades, outcome remains fatal in approximately one third of patients. Primary chemoresistance, relapse and acute and long-term toxicities to conventional myelosuppressive therapies still constitute significant challenges and emphasize the unmet need for effective targeted therapies. Years of scientific efforts have translated into extensive insights on the heterogeneous spectrum of genetics and oncogenic signaling pathways of AML and identified a subset of patients characterized by upregulation of *HOXA* and *HOXB* homeobox genes and *myeloid ecotropic virus insertion site 1* (*MEIS1*). Aberrant *HOXA*/*MEIS1* expression is associated with genotypes such as rearrangements in *Histone-lysine N-methyltransferase 2A* (*KMT2A*-r*)*, *nucleoporin 98* (*NUP98*-r) and mutated *nucleophosmin* (NPM1c) that are found in approximately one third of children with AML. AML with upregulated *HOXA*/*MEIS1* shares a number of molecular vulnerabilities amenable to recently developed molecules targeting the assembly of protein complexes or transcriptional regulators. The interaction between the nuclear scaffold protein menin and KMT2A has gained particular interest and constitutes a molecular dependency for maintenance of the *HOXA*/*MEIS1* transcription program. Menin inhibitors disrupt the menin-KMT2A complex in preclinical models of *KMT2A*-r, *NUP98*-r and NPM1c acute leukemias and its occupancy at target genes leading to leukemic cell differentiation and apoptosis. Early-phase clinical trials are either ongoing or in development and preliminary data suggests tolerable toxicities and encouraging efficacy of menin inhibitors in adults with relapsed or refractory *KMT2A*-r and NPM1c AML. The Pediatric Acute Leukemia/European Pediatric Acute Leukemia (PedAL/EUPAL) project is focused to advance and coordinate informative clinical trials with new agents and constitute an ideal framework for testing of menin inhibitors in pediatric study populations. Menin inhibitors in combination with standard chemotherapy or other targeting agents may enhance anti-leukemic effects and constitute rational treatment strategies for select genotypes of childhood AML, and provide enhanced safety to avoid differentiation syndrome. In this review, we discuss the pathophysiological mechanisms in *KMT2A*-r, *NUP98*-r and NPM1c AML, emerging molecules targeting the *HOXA/MEIS1* transcription program with menin inhibitors as the most prominent examples and future therapeutic implications of these agents in childhood AML.

## 1. Introduction

Acute myeloid leukemia (AML) is a highly aggressive hematological malignancy characterized by rapid proliferation of leukemic blasts leading to impaired normal hematopoiesis. AML is the most frequent acute leukemia in adults but relatively rare in children with an occurrence of only ~8 per million children annually (Puumala et al., 2013). Intensified treatment schedules including retrieval at relapse, optimization of hematopoietic stem cell transplantation (SCT), and improved supportive care have gradually improved event-free and overall survival (EFS/OS) of pediatric AML to approximately 60 and 75% (Creutzig et al., 2013; Gamis et al., 2014; Hasle et al., 2012; Rubnitz et al., 2010). Nevertheless, several challenges remain: 1) further intensification of myelosuppressive therapy in order to decrease the risk of relapse will likely be counterbalanced by excess toxicities and aggravate current rates of treatment-related mortality (Creutzig et al., 2004; Klein et al., 2020) and intolerable short- and long-term morbidities (Bhatt et al., 2021; Løhmann et al., 2016; Mulrooney et al., 2008; Sørensen et al., 2019). 2) Frequencies of refractory disease and relapse have only slightly decreased or remained constant for several years (Alexander et al., 2017; Rasche et al., 2018). 3) Outcome after relapse remains dismal in selected genetic subgroups with high rates of refractory disease, treatment-related deaths and second relapse (Karlsson et al., 2017; Rasche et al., 2021). Thus, an unmet need for novel and improved treatment modalities for children with AML remains.

Through the last decade, high throughput sequencing technologies have elucidated the heterogeneous genomic landscape of AML and have identified significant differences in cytogenetic and molecular aberrations between pediatric and adult age groups (Bolouri et al., 2018; The Cancer Genome Atlas Research Network, 2013). These data have not only informed disease and risk classification systems but also generated a growing interest in precision medicine, i.e. therapies targeting leukemia-specific pathways and with limited effects on non-malignant cell homeostasis. Some of the most prominent examples of targeted therapies in AML are inhibitors of the FMS-like tyrosine kinase (FLT3) which is recurrently mutated by internal tandem duplications (ITD) in up to 25% of adults (Perl et al., 2017; Stone et al., 2017). Even though FLT3 inhibition may also ameliorate the poor prognosis of *FLT3*-ITD AML in children (Pollard et al., 2022), the proportion of children with this target is far less predominant compared to adults. Likewise, enrollment from adult AML populations has advanced therapies such as ivosidenib and inasidenib targeting *IDH 1/2* which are detected in less than 5% of children (Andersson et al., 2011) illustrating the relative scarcity of pediatric AML patients currently applicable for biomarker-driven treatment approaches.

### 1.1. HOX and MEIS1 expression in hematopoiesis and leukemia

Hematopoiesis is governed by a complex hierarchy of transcription factors that regulate the expression of multiple genes across various states of differentiation (Novershtern et al., 2011). One of the most evolutionarily conserved gene families encoding such transcription factors are the *HOX* homeobox genes. The *HOX* genes are organized in clusters (A-D) and critical for embryogenesis and stem cell differentiation in various tissues (Seifert et al., 2015). *HOX* genes, particularly *HOXA*, *HOXB* clusters, and the *HOXA* co-factor *myeloid ecotropic virus insertion site 1* (*MEIS1*) are well-characterized regulators of self-renewal properties and differentiation of myeloid progenitors. Expression of *HOXA/B* and *MEIS1* genes is high in immature stem cell populations of the bone marrow and decreases during maturation and differentiation of hematopoiesis (Giampaolo et al., 1994; Pineault et al., 2002; Sauvageau et al., 1994). The critical role of these genes in hematopoiesis is supported by observations of deleterious effects on myelopoiesis including reduction of functional peripheral blood leukocytes and platelets in animals with loss-of-function mutations in *HOXA* genes and *MEIS1* (Hisa et al., 2004; Konieczna et al., 2016; Lawrence et al., 1997). In contrast, overexpression of *HOXA/MEIS1* confers proliferative advantages to hematopoietic cells, perturbs differentiation and ultimately leads to leukemic transformation (Bach et al., 2010; Thorsteinsdottir et al., 1997, 2001). A stem-cell-like expression of *HOXA*/*MEIS1* is a hallmark of several genetic subtypes, that are prevalent in pediatric AML patients and include aberrations such as rearrangements in *Histone-lysine N-methyltransferase 2A* (*KMT2A*-r*)* (Armstrong et al., 2002; Ferrando et al., 2003; Kawagoe et al., 1999), mutated *nucleophosmin* (*NPM1*) and rearrangements in *nucleoporin 98* (*NUP98*-r) (Alcalay et al., 2005; Bisio et al., 2017; Hollink et al., 2011; Mullighan et al., 2007). The biological processes leading to aberrant *HOX*/*MEIS1* expression are complex but accumulating data suggests that the interaction between the KMT2A complex and the chromatin-associated scaffold protein menin plays a pivotal role in activating a leukemic transcription program (Caslini et al., 2007; Chen et al., 2006; Hughes et al., 2004; Yokoyama et al., 2005). In the context of pediatric AML, the recent development of small-molecule inhibitors targeting the menin-KMT2A interaction is of particular interest as rearrangements in *KMT2A* are some of the most common aberrations. Furthermore, menin inhibition may also restore normal *HOXA*/*MEIS1* expression in mutated *NPM1* and *NUP98*-r AML and thus have therapeutic implications in up to one third of pediatric AML patients, of whom a significant proportion have high-risk disease incurable with current treatment modalities. This review summarizes the clinical and molecular characteristics of genetic entities with aberrant *HOXA/B/MEIS1*, agents targeting the menin-KMT2A interaction and other molecular vulnerabilities in assembly of protein complexes or general transcription machinery as well as implications of these evolving therapies in childhood AML.

### 1.2. Genotypes with aberrant HOXA/MEIS expression

#### 1.2.1. AML with KMT2A rearrangements

*KMT2A*-rearrangements are not only restricted to leukemia of myeloid lineage. *KMT2A* (also known as *MLL*) is also recurrently rearranged in acute lymphoblastic leukemia (ALL) and mixed phenotype acute leukemia (MPAL) and especially infant ALL is characterized by a high incidence of *KMT2A* translocations in up to 75% of patients (Pieters et al., 2019). Altogether, 10% of children with acute leukemias harbor rearrangements in *KMT2A,* a proto-oncogene capable of fusing with more than 100 different partner genes or undergoing partial internal tandem duplication to transform into a potent oncogene (Meyer et al., 2023). With the exception of previous exposure to cytotoxic agents, in particular epipodophyllotoxins (Pui et al., 1991), no risk factors for development of *KMT2A*-r AML have been identified. Rearrangements in *KMT2A* are present in approximately 20% of children with de novo AML (Harrison et al., 2010; Neuhoff et al., 2010) and particularly frequent in young children where 40% of newly diagnosed patients are less than 2 years of age (Balgobind et al., 2009; Bolouri et al., 2018; Creutzig et al., 2016). High white blood cell count, persistence of measurable residual disease (MRD) during induction therapy, age above 10 years and additional cytogenetic aberrations are all associated with an inferior outcome in children with *KMT2A*-r AML (Balgobind et al., 2009; Pollard et al., 2021; Weelderen et al., 2023), though chromosomal fusion partner is considered the most important predictor of outcome. The vast majority of *KMT2A* translocations are associated with an intermediate or inferior prognosis (Table 1). Patients with *KMT2A*::*MLLT11* and *KMT2A*::*MLLT3* fusions have previously demonstrated superior outcomes with OS of 100% and 65% respectively (Balgobind et al., 2009; Rubnitz et al., 2002). However, more recent data suggests that prognosis in these patients are comparable to other intermediate-risk fusion partners (Pollard et al., 2021). Response to induction therapy assessed both by morphology and flow cytometry is comparable across *KMT2A* fusion types, however, one third of the patients with high-risk fusions such as *KMT2A*::*AFF1*, *KMT2A*::*MLLT4*, and *KMT2A*::*MLLT10* are characterized by high rates of disease recurrence with only limited chance of salvage and consequently dismal OS (Table 1) (Balgobind et al., 2009; Pollard et al., 2021; Weelderen et al., 2023).

*KMT2A*-rearrangements are potent oncogenic drivers and characterized by a paucity of co-occurring mutations in both adults and children (Bolouri et al., 2018; Lavallée et al., 2015). RAS pathway mutations (*NRAS*, *KRAS, PTPN11 or NF1*) may be present in up to 30% of children with *KMT2A*-r AML but seemingly these secondary events show no significant impact on prognosis (Balgobind et al., 2011; Berman et al., 2011; Bolouri et al., 2018).

**Fig. 1.**
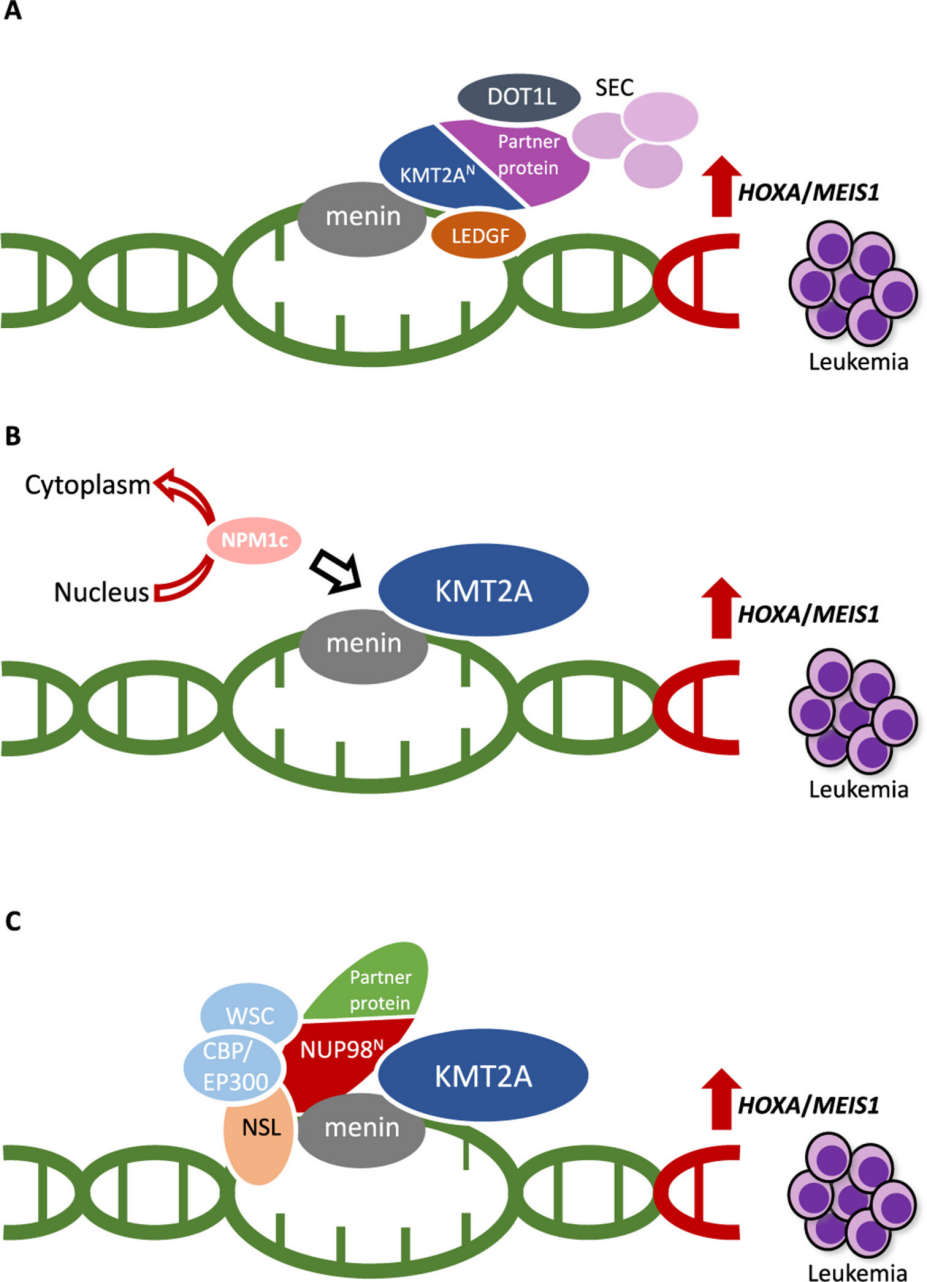
Leukemogenic mechanisms in AML with *KMT2A*-rearrangements (A), mutated *NPM1* (B) or *NUP98* rearrangements (C). Menin-KMT2A interaction is critical for the oncogenic machinery of *HOXA*/*MEIS1* deregulation in all three genotypes. CBP/EP300, cyclic adenosine monophosphate response element-binding protein/E1A-binding protein of p300; DOT1L, disruptor of telomere silencing 1; KMT2A, Histone-lysine N-methyltransferase 2A; LEDGF, Lens Epithelium-Derived Growth Factor; NSL, non-specific lethal histone-modifying complex; SEC, super elongation complex; WSC, Wdr82–Set1A/COMPASS.

KMT2A is an approximately 500 kDa nuclear protein that is activated by taspase-1-mediated cleavage to an N-terminal 320 and a C-terminal 180 kDa fragment. The dimer acts as an epigenetic modulator of several target sequences including *HOXA* genes (Guenther et al., 2005; Milne et al., 2005). The C-terminus of the wild-type protein contains a number of domains such as PHD-fingers, bromodomain, activation domain and SET domain, that mediate histone binding and protein interactions critical for both transcriptional repression and activation of KMT2A target genes (Chen et al., 2008; Zeleznik-Le et al., 1994). Rearrangement of the *KMT2A* gene implies loss of the wild-type C-terminus, and alternately generation of a fusion protein with a different C-terminus partner leading to impaired target gene regulation. Despite diverse functions and structures of the various partner proteins, KMT2A fusions share distinct chromatin-binding profiles and interact with various protein complexes such as the super elongation complex (SEC), the polymerase-associated factor complex (PAFc), and disruptor of telomere silencing 1 (DOT1L), constitutively enhancing *HOXA* and *MEIS1* transcription (Bernt et al., 2011; Lin et al., 2010; Muntean et al., 2010; Okada et al., 2005; Okuda et al., 2017; Xu et al., 2016a, b) (Fig. 1A). The N-terminus portion of KMT2A is retained in all fusion proteins including the menin binding site, a domain of major functional importance that links to a well-characterized, high affinity site on menin (Murai et al., 2011). Interactions between menin, KMT2A fusion protein and the chromatin-associated protein Lens Epithelium-Derived Growth Factor (LEDGF) are critical for malignant transformation as disruption of these protein interactions leads to loss of the *HOXA/MEIS1* transcription program (Ashkar et al., 2017; Caslini et al., 2007; Chen et al., 2006; Yokoyama et al., 2005; Yokoyama and Cleary, 2008; Zhu et al., 2016).

#### 1.2.2. AML with mutated NPM1

*NPM1* is recurrently mutated in AML and the most frequent molecular alteration in adult-onset disease (Papaemmanuil et al., 2016). In children, *NPM1* mutations are present in approximately 8% of the patients and with a female predominance. *NPM1* mutations are rare in young children with AML but show an increasing frequency according to age, particularly in patients with a normal karyotype, where *NPM1* is mutated in 20–30 % of cases (Brown et al., 2007; Cazzaniga et al., 2005; Hollink et al., 2009). In general, *NPM1* mutations portend a favorable prognosis with rates of EFS and OS exceeding 70 and 80 % respectively (Xu et al., 2020). *FLT3-*ITD is associated with an adverse outcome and present in up to one third of children with mutated *NPM1*, but limited study populations hamper the establishment of prognostic interaction between concomitant *FLT3*-ITD and *NPM1* mutations (Brown et al., 2007; Hollink et al., 2009). In the most recent cohort study including 869 children with AML from the TARGET data set, *NPM1* mutation was associated to a superior outcome regardless of *FLT3*-ITD status and constituted an independent predictor of a favorable prognosis (Xu et al., 2020).

NPM1 is a molecular chaperone with multiple biological functions including transport of ribosomal components between the nucleus and cytoplasm, preservation of DNA repair and stability and regulation of apoptotic pathways (Poletto et al., 2014; Vascotto et al., 2014; Yu et al., 2006). Tetranucleotide insertions in exon 12 of the *NPM1* gene (type A and B) are the most frequent mutation subtypes (Brown et al., 2007; Hollink et al., 2009) and lead to truncation of the protein and aberrant cytoplasmic localization of NPM1 (NPM1c) (Falini et al., 2005). This in turn results in an aberrant localization of NPM1c-associated nuclear proteins into the cytoplasm, including the protein PU.1, a driver of monocyte lineage differentiation. Loss of PU.1 from the nucleus in NPM1c AML switches the function of nuclear transcription factors CCAAT/enhancer binding protein alpha (C/EBPα) and Runt-related transcription factor 1 (RUNX1*)* from activation to suppression of more than 500 terminal differentiation genes (Gu et al., 2018). The suppression of differentiation by NPM1c results in upregulation of *HOXA, HOXB* and *MEIS1* gene expression (Alcalay et al., 2005; Brunetti et al., 2018; Spencer et al., 2015) (Fig. 1B). The exact pathways leading to aberrant expression of *HOXA, HOXB and MEIS1* by the relative absence of nuclear NPM1/PU.1 and/or toggle of associated transcription factors are not fully elucidated. Nonetheless, a number of recent preclinical investigations demonstrate that the leukemic transcription program is activated by NPM1c chromatin-binding to target gene promoters and highly dependent on wild-type KMT2A-menin interaction as either genomic editing or pharmacological inhibition of menin suppress pro-leukemic gene expression and lead to hematopoietic differentiation in NPM1c AML models (Klossowski et al., 2019; Kühn et al., 2016; Uckelmann et al., 2020, 2022; Wang et al., 2022).

#### 1.2.3. AML with NUP98 rearrangements

NUP98 rearrangements may occur across the spectrum of hematological malignancies including AML, myelodysplastic syndrome, chronic myeloid leukemia, ALL and MPAL (Mitelman et al., 2022). Owing to the sub-telomeric location of breakpoint, *NUP98-r* AML is commonly cryptic and may not be recognized by conventional karyotyping. Immunofluorescence and molecular techniques identify *NUP98*-r in 5–10% of children with AML, with more than 30 different partner genes identified to date (Bisio et al., 2017; Hollink et al., 2011; Struski et al., 2017). The genes encoding the nuclear receptor binding SET-domain protein 1 (*NSD1*) and Lysine-specific demethylase 5A (*KDM5A;* formerly known as *JARID1A*) are the two most common translocation partners in childhood *NUP98*-r AML and both have distinct molecular and disease characteristics. While NSD1 methylates lysine 36 of histone 3, KDM5A demethylates lysine 4 of the same histone. *NUP98::NSD1* fusions are virtually absent in children younger than 2 years of age and often present with FAB-M4/M5 morphology and pronounced hyperleukocytosis (Hollink et al., 2011; Struski et al., 2017). In contrast, children with *NUP98::KDM5A* fusions are predominantly small children (less than 4 years of age) and diagnosed with acute megakaryoblastic leukemia (FAB M7) (Noort et al., 2020). As a whole, children with *NUP98*-r AML are considered an entity with high-risk disease, even though outcome measures of rare *NUP98* fusion partners are inadequately characterized. Strikingly, *NUP98::NSD1* presents as the only chromosomal abnormality in the majority of cases, but is accompanied by *FLT3*-ITD and/or mutations in the Wilms tumor gene (*WT1*) in more than 80% of cases. The interaction of these high-risk aberrations may lead to especially high rates of primary refractory disease and early relapses in children with *NUP98::NSD1* AML and dismal long-term survival (Bisio et al., 2017; Hollink et al., 2011; Niktoreh et al., 2019; Struski et al., 2017). However, the lack of a clinically significant benefit from FLT3-inhibitors in *NUP98::NSD1*/*FLT3*-ITD AML indicates that the NUP98 fusion is a potent leukemic driver with an independent impact on prognosis (Tarlock et al., 2019). Even though *NUP98::KDM5A* fusions do not seem to further deteriorate the poor prognosis of FAB M7 AML (Rooij et al., 2013), unselected childhood AML patients with these fusions show inferior and superimposable EFS and OS indicating only minimal chance of salvage after relapse (Noort et al., 2020) (Table 1).

The precursor protein encoded by the *NUP98* gene is proteolytically cleaved into 98 kDa and 96 kDa nucleoporins that both exert important functions in maintenance of cell homeostasis and renewal capacity. NUP98 is a component of the nuclear pore complex (NPC), a multiprotein structure situated in the nuclear envelope facilitating bidirectional transport of various molecules between the cytoplasm and nucleus (Fontoura et al., 1999; Griffis et al., 2003). The N-terminal of NUP98 contains two rows of non-tandem amino acid repeats (collectively termed FG repeats – 38 in total) that both serve as binding sites for the karyopherins, carrier proteins of the NPC that mediate the active transport of macromolecules across the nuclear membrane (Radu et al., 1995), but also constitute residues of interaction with hematopoietic transcriptional regulators such as cyclic adenosine monophosphate response element-binding protein/E1A-binding protein of p300 (CBP/EP300) and Wdr82–Set1A/COMPASS (WSC) (Franks et al., 2017; Kasper et al., 1999). The FG repeats are bisected by the Gle2-binding sequence (GLEBS) which is critical for RNA transport through the NPS mediated by the RNA export factor RAE1, but also plays a pivotal role in mitotic spindle and cell cycle regulation (Jeganathan et al., 2005; Ren et al., 2010).

While the C-terminal is replaced by the partner protein, the N-terminus of NUP98 is retained in all NUP98 fusion proteins. The relative contribution of the fusion protein moieties to leukemogenesis is a matter of debate and may vary between the individual fusion partners. SET and PHD finger domains of the fusion partners *NSD1* and *KDM5A* have chromatin-modifying capacity and prevents transcriptional repression of several target genes including *HOXA/B* genes and *MEIS1* (Wang et al., 2007, 2009). Although the wild-type NUP98 protein is mainly found within the NPC, NUP98 fusions are mostly localized within the intranuclear clusters known as “GLFG” bodies across the nucleoplasm, where they facilitate an increased engagement between N-terminal FG repeats and transcriptional regulators in the nucleoplasm. Notably, interaction between NUP98 fusion protein, the non-specific lethal (NSL) histone-modifying complex and KMT2A-menin is deemed essential for induction of leukemia and aberrant *HOXA, HOXB* and *MEIS1* transcription (Rio-Machin et al., 2017; Shima et al., 2017; Xu et al., 2016a, b) (Fig. 1C).

### 1.3. Menin inhibition

#### 1.3.1. Preclinical studies

Menin is encoded by the *MEN1* gene and constitutional loss of its tumor suppressor function may lead to endocrine hyperplasia or neoplasms known as the multiple endocrine neoplasia type 1 syndrome. However, menin also displays oncogenic properties and the interaction with the KMT2A protein is deemed critical for maintenance of the leukemic transcription program in AML genotypes with *HOXA*/*MEIS1* expression. A number of orally available small-molecule inhibitors that bind with high affinity to the KMT2A binding pocket of menin have been developed and optimized over the past decade. In preclinical models of *KMT2A*-r leukemia these molecules disrupt the menin-KMT2A interaction and inhibit the chromatin occupancy of menin and KMT2A at select KMT2A target genes leading to pronounced suppression of predominantly *MEIS1*, but also *HOXA* genes. These inhibitors demonstrate selective responses in *KMT2A*-r cell lines and robust and dose-dependent single-agent activity in xenograft models without detrimental effects on normal hematopoiesis (Borkin et al., 2015; Grembecka et al., 2012; Klossowski et al., 2019; Krivtsov et al., 2019). Despite differences in transformation capacity between fusion partners, menin inhibition leads to the eradication of disease and profound survival benefits across various *KMT2A* fusion types (He et al., 2016; Krivtsov et al., 2019).

In both cell lines and patient-derived NPM1c leukemia cells menin inhibition removes the menin-KMT2A complex from target gene loci and suppresses expression of especially *MEIS1* and its transcriptional target *FLT3*. In NPM1c PDX models, menin inhibition shows phenotypic effects very similar to observations from *KMT2A*-r models and leads to terminal differentiation, leukemic cell death and prolonged survival (Klossowski et al., 2019; Kühn et al., 2016; Uckelmann et al., 2020).

The NUP98 fusion forms complex with multiple proteins and recently the pivotal role of the interaction between NUP98 fusions and menin-KMT2A was demonstrated in mouse models. Menin inhibition in vitro leads to loss of chromatin occupancy of NUP98 fusion complexes and consequently *MEIS1* downregulation. In vivo studies confirmed upregulation of markers of differentiation and dramatic anti-leukemic effects leading to prolonged survival in PDX animals (Heikamp et al., 2022). The dependency of the functional menin-KMT2A interaction has also been demonstrated in ex vivo studies using primary samples of *NUP98*::*NSD1* positive AML where menin inhibition induced myeloid differentiation linked with impaired proliferation and clonogenicity (Rasouli et al., 2021).

#### 1.3.2. Clinical studies

Definitive preclinical data from acute leukemia genotypes with *HOXA*/*MEIS1* upregulation suggests promising anti-leukemic activity of menin inhibition without deteriorating effects on normal hematopoiesis and has paved the way for a number of first-in-human clinical trials (Table 2).

AUGMENT-101 (NCT04065399) is an ongoing phase I/II, dose-escalation and expansion study of single-agent revumenib (formerly named SNDX-5613) in adults with relapsed/refractory (R/R) acute leukemia. Revumenib is a substrate of CYP3A4 and thus, pharmacokinetics (PK), safety and efficacy of the drug are investigated both with and without concomitant CYP3A4 inhibitors (i.e. azoles). As of March 2022, 68 patients were enrolled across 4 dose levels in the phase I portion of the trial. Revumenib was well tolerated and with no discontinuations due to treatment-related adverse events (AE). Asymptomatic ECG QTc prolongation was the dose-limiting toxicity and the only grade 3 or greater treatment-related AE present in at least 5% of the patients (13%). In the efficacy population with either *KMT2A*-r (the majority being AML) or NPM1c leukemias, 32/60 patients (53%) achieved an overall response (composite endpoint of CR, CR with partial hematologic recovery, CR with incomplete platelet recovery and morphologic leukemia-free state). Among the 32 responders, 18 patients (56%) obtained MRD negativity and 12 (38%) proceeded to SCT (Issa et al., 2022, 2023).

To date, experience with revumenib in children is scarce. The AUGMENT-101 was amended in 2020 to include children 30 days or older and is now open in both North America and Europe. Children at various ages have received revumenib monotherapy both on AUGMENT-101 as well as through the FDA compassionate use program and data on safety, PK and efficacy are pending. A recently initiated phase I trial, the AUGMENT-102 (NCT05326516), will provide confidence in pediatric dosing of revumenib in combination with chemotherapy as it includes both children and adults with R/R *KMT2A*-r, *NUP98*-r and NPM1c acute leukemias and aims to determine the safety and tolerability of revumenib in combination with standard myeloid and lymphoid chemotherapy backbones (Table 2). Furthermore, safety and efficacy of revumenib in combination with decitabine/cedazuridine (ASTX727) and venetoclax is currently investigated in the SAVE trial (NCT05360160), that also includes children more than 12 years of age with R/R AML unfit for intensive chemotherapy (Table 2).

The KOMET-001 (NCT04067336) is a phase I/II dose-escalation and expansion trial evaluating the safety, tolerability and efficacy of ziftomenib monotherapy in adult R/R acute leukemias. As of October 2022, 30 patients had been treated with ziftomenib across 7 dose levels in the phase Ia portion of the trial. Anemia, pneumonia, neutropenia, thrombocytopenia, febrile neutropenia and decreased appetite categorized as a grade 3 or greater treatment-emergent AE were observed in at least 10% of the patients. There has been no evidence of QTc prolongation or other ECG changes and preliminary PK analyses suggest no significant CYP3A4 interaction. In the ongoing phase Ib portion of the trial, dose levels of 200 mg and 600 mg are evaluated in patients with *KMT2A*-r or NPM1c AML in order to identify the optimal biologically active dose. Grade 3 or greater differentiation syndrome developed in 8/53 patients in the phase Ib cohort (15%) and was potentially associated to a fatal AE in one patient, which led to a temporary, partial clinical hold in November 2021 and revised mitigation strategies (Kura Oncology, 2022). Response evaluation shows a dose-dependent efficacy signal of ziftomenib and an overall response rate of 29% (11/38) in patients treated at the 600 mg dose level (Erba et al., 2022). A phase Ia/Ib trial of ziftomenib in combination with chemotherapy regimens of both low and high intensities are currently in development and planned to open for recruitment of adults with either newly diagnosed or R/R *KMT2A*-r or NPM1c AML in 2023 (NCT05735184).

The food effects, safety and tolerability of JNJ-75276617, a menin inhibitor from Janssen Pharmaceuticals, is being evaluated in a phase I trial inclusive of adults with R/R *KMT2A*-r and NPM1c acute leukemias (NCT04811560), and will also be evaluated in a combination regimen with venetoclax or/and azacytidine (NCT05453903). A pediatric formulation of JNJ-75276617 is available and a company-sponsored combination trial of R/R acute leukemias in children and young adults up to 30 years of age is in development (NCT05521087). Biomea Fusion, Daiichi Sankyo and Sumitomo Dainippon Pharma have also initiated early phase clinical trials with menin inhibitor therapies for adult-onset leukemias (Table 2).

The Pediatric Acute Leukemia/European Pediatric Acute Leukemia (PedAL/EUPAL) project, a collaborative research initiative as an offspring of the ACCELERATE AML platform meeting in 2019 (Pearson et al., 2020), and supported by the Leukemia Lymphoma Society, is actively engaged with multiple biopharma companies to develop and conduct studies with menin inhibitors focused on pediatric acute leukemias (ACCELERATE virtual meeting on Menin inhibitors, 2022).

### 1.4. Other targeted therapies

**Fig. 2.**
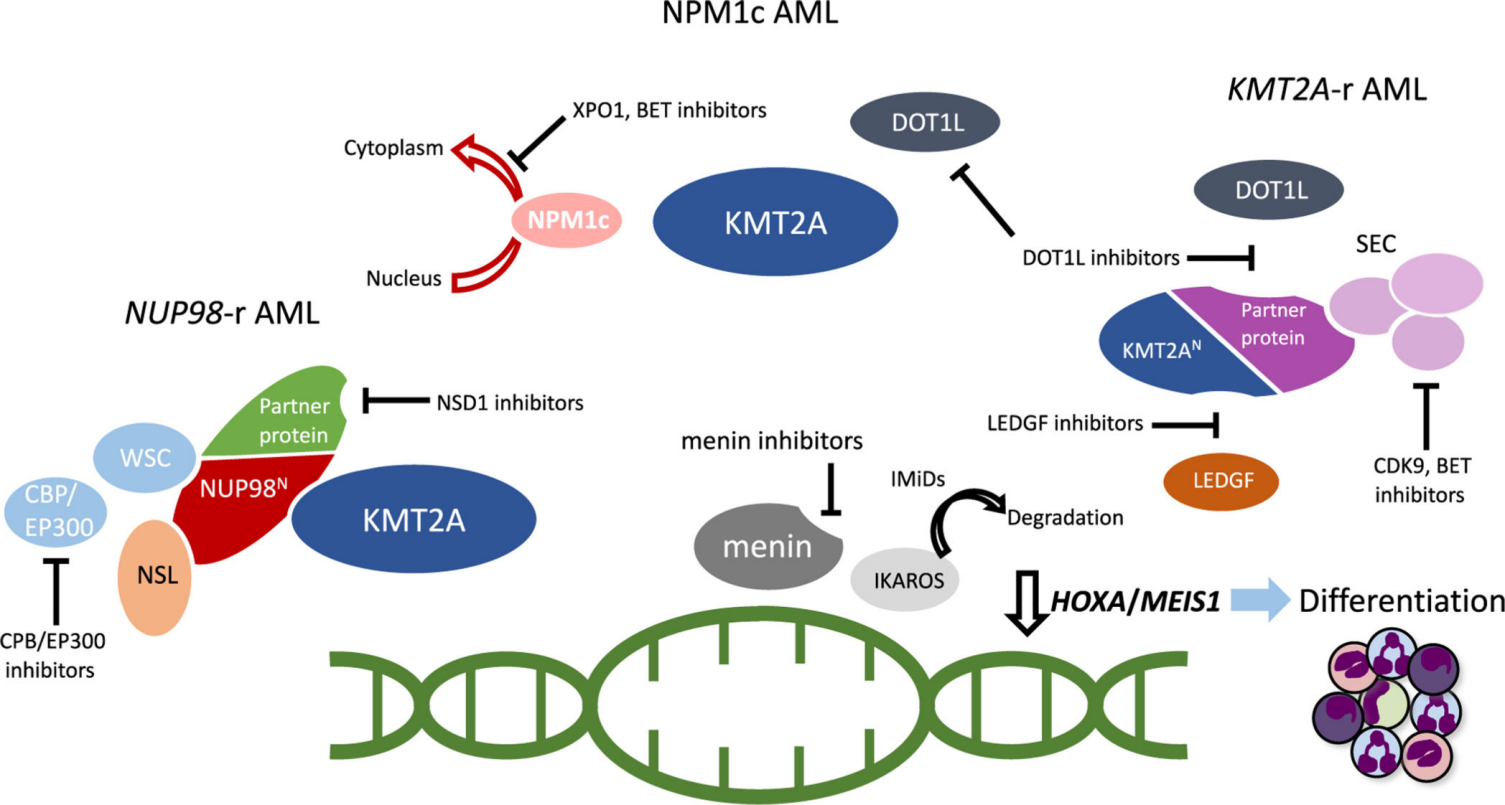
Various mechanisms of action of molecules targeting the assembly of protein complexes or transcriptional regulation in AML with *KMT2A*-rearrangements, mutated *NPM1* and *NUP98* rearrangements. BET, bromodomain and extra terminal family of proteins; CBP/EP300, cyclic adenosine monophosphate response element-binding protein/E1A-binding protein of p300; CDK9, cyclin-dependent kinase 9; DOT1L, disruptor of telomere silencing 1; KMT2A, Histone-lysine N-methyltransferase 2A; IMiDs, Immunomodulatory imide drugs; LEDGF, Lens Epithelium-Derived Growth Factor; NSD1, nuclear receptor binding SET-domain protein 1; SEC, super elongation complex; WSC, Wdr82–Set1A/COMPASS; XPO1, Exportin 1.

Combination of menin inhibitors with contemporary chemotherapy regimens may provide additive anti-leukemic effects but the addition of agents directed at other molecular targets than the menin-KMT2A interaction may as well be an appealing strategy to obtain therapeutic synergy in select genotypes. These agents may include inhibitors disrupting the assembly of protein complexes, or alternately molecules interfering with the transcription machinery (Fig. 2). Pharmacological inhibition of DOT1L in cell line and xenograft models of *KMT2A*-r leukemia has shown anti-leukemic activity and restores normal *HOXA* and *MEIS1* transcription (Daigle et al., 2011, 2013). Complementary cell killing activity and more profound epigenetic regulation may be obtained by combined menin and DOT1L inhibition and synergy of these molecules has been observed in preclinical models of both *KMT2A*-r and NPM1c AML (Dafflon et al., 2017; Kühn et al., 2016). The DOT1L inhibitor pinometostat showed only modest clinical activity as monotherapy in adult patients with *KMT2A*-r acute leukemias, though the maximum tolerated dose of the agent was not reached (Stein et al., 2018). Thus, optimal dosing and potential benefit of combined DOT1L and menin targeting is still rational to explore in future clinical trials.

Another target of interest is the interaction between the menin-KMT2A complex and the transcriptional co-activator LEDGF. Inhibitors of LEDGF selectively impair leukemic cell growth without detrimental effects on normal hematopoiesis in preclinical models of *KMT2A*-r leukemia (Ashkar et al., 2017; Méreau et al., 2013; Murai et al., 2014) but have currently not advanced to testing in clinical trials.

In AML harboring the NUP98:NSD1 fusion protein, the leukemogenic transcription program depends on the catalytic activity of the NSD1 SET domain. A newly developed, covalent inhibitor of the NSD1 SET domain selectively impairs proliferation and reduces *HOXA* and *MEIS1* expression in mouse and human *NUP98*::*NSD1* positive cell lines (Huang et al., 2020). Further development and optimization in the preclinical setting is ongoing, before this promising class of molecules can be advanced into clinical trials.

Pharmacological inhibition of transcriptional regulators is widely applicable in cancer therapy, including various hematological malignancies. In leukemia patients with NPM1c, or rearrangements in *KMT2A* and *NUP98*, a number of these regulators are considered instrumental for loss of transcriptional control and aberrant expression of *HOXA/B/MEIS1*, and other leukemia-associated target genes. The cyclin-dependent kinase 9 (CDK9), a component of the positive transcription elongation factor b (P-TEFb) and SEC, enables transcription elongation through activation of the RNA polymerase II. CDK9 inhibitors are potent inducers of apoptosis (Baker et al., 2016; Cidado et al., 2020). The second generation CDK2/CDK9 inhibitor, fadraciclib, has significant anti-leukemic effects in xenograft models of *KMT2A*-r AML (Chantkran et al., 2021; Frame et al., 2020) and is currently tested in combination with venetoclax in a phase I trial of adult R/R AML (NCT04017546).

Bromodomain-containing protein 4 (BRD4), a member of the bromodomain and extra terminal (BET) family of proteins, is an epigenetic reader and aberrantly activated by interaction with SEC and PAFc or relative absence of NPM1 in the nucleoplasm. BET-inhibitors are effective in eliminating leukemia cells and suppressing target gene transcription in AML mouse models including those for *KMT2A*-r and NPM1c AML (Dawson et al., 2011, 2014; Zuber et al., 2011). Certain classes of BET inhibitors may also target the bromodomain of CBP/EP300, the transcriptional coactivator recruited by both KMT2A and NUP98 fusion proteins (Imayoshi et al., 2021; Picaud et al., 2015; Spriano et al., 2020). Recently, combined BET and menin targeting has demonstrated synergistic lethality in *KMT2A*-r and NPM1c AML cell lines (Fiskus et al., 2022b). Two clinical trials are currently testing the safety of the BET-inhibitor BMS-986158 in children with advanced solid tumors and hematological malignancies (NCT02419417; NCT03936465).

Recently IKAROS, a transcription factor encoded by the *IKZF1* gene, was identified as an important transcriptional regulator that co-occupies chromatin with the menin-KMT2A complex in *KMT2A*-r and NPM1c AML. Immunomodulatory imide drugs (IMiDs), which have FDA approval for treatment of multiple myeloma and myelodysplastic syndrome, degrade IKAROS and disrupt the expression of *HOXA9* target genes without affecting *HOXA9* expression itself. IMiD-mediated degradation of IKAROS sensitized leukemia cells to treatment with both menin and DOT1L inhibitors, and combination of IMiDs and menin inhibition induced apoptosis in *KMT2A*-r and NPM1c cell lines, intensified the transcriptional changes and prolonged survival in xenograft mice with human *KMT2A*-r leukemia compared to monotherapy (Aubrey et al., 2022).

*HOXA*/*MEIS1* expression and loss of differentiation in NPM1c AML is driven by the nuclear export of NPM1 and mislocalization to the cytoplasm. Inhibition of Exportin 1 (XPO1), a nucleocytoplasmic shuttling protein of the karyopherin family, retains NPM1 within the nucleus, repress *HOX* and *MEIS1* expression and enables terminal monocytic differentiation in mice (Brunetti et al., 2018). XPO1 inhibitors are approved by the FDA for multiple myeloma and demonstrated an acceptable toxicity profile, but only modest clinical activity as monotherapy in a phase I study of adult all-comers with R/R AML (Garzon et al., 2017). However, a more frequent dosing schedule leading to a sustained loss of the XPO1-NPM1c interaction may optimize the anti-leukemic activity of XPO1 inhibition therapy in NPM1c AML (Pianigiani et al., 2022). Both preclinical and early phase clinical data suggest that XPO1 inhibitors may elicit additive anti-leukemic activity when combined with established or targeted therapies (Ranganathan et al., 2016; Sweet et al., 2020; Zhang et al., 2018), and synergize with menin inhibition in NPM1c AML (Uckelmann et al., 2022). The maximum tolerated dose of the XPO1 inhibitor selinexor in combination with FLA has been established in children with R/R acute leukemias (Alexander et al., 2016) and a pediatric trial investigating a combination regimen of selinexor and venetoclax with or without chemotherapy is currently ongoing (NCT04898894).

Rearrangements in *KMT2A*, *NUP98* and mutated *NPM1* are considered potent drivers of leukemia but may be accompanied by activation of other oncogenic pathways of both prognostic and therapeutic relevance. It is outside the scope of this review to provide an extensive overview of co-mutational strategies, however, menin inhibitors together with inhibitors of FLT3 activating mutations or B-cell lymphoma 2 (BCL-2) protein may be highlighted as promising combination therapies of particular relevance considering the observations of synergy in preclinical models (Carter et al., 2021; Dzama et al., 2020; Fiskus et al., 2022a; Miao et al., 2020).

### 1.5. Perspectives

Despite decades of intensive research on genetic aberrations and leukemia ontogeny, only a few leukemia-specific targets applicable for therapeutic intervention have been identified in pediatric AML. The menin-KMT2A interaction promotes aberrant *HOXA*/*MEIS1* expression across various recurrent AML genotypes and has emerged as a prominent molecular dependency that may be disrupted by a new class of selective molecules. In addition to mutated *NPM1* and rearrangements of *KMT2A* and *NUP98*, other genetic subsets are characterized by elevated *HOXA/B* gene cluster or *MEIS1* levels (systematically summarized by Issa et al., 2021). For instance, rare pediatric AML cases with *PICALM*::*MLLT10* or *DEK*::*NUP214* fusions have very poor outcomes and are characterized by increased expression of *HOXA* and *MEIS1* (Novak et al., 2012; Ries et al., 2019; Sandahl et al., 2014).

In preclinical studies, *MEIS1* expression appears to be a responsive biomarker (Heikamp et al., 2022; Klossowski et al., 2019; Krivtsov et al., 2019) and may constitute an informative screening tool to identify patients who may benefit from menin inhibition or other therapies targeting the *HOXA/B*/*MEIS1* transcription program. However, validated assays to reproducible quantify *HOXA/B* and *MEIS1* expression in the clinical setting and differentiate abnormally high levels from background expression in normal hematopoiesis are lacking. Furthermore, recent investigations suggest that low *HOXA*/*MEIS1* expression may not necessarily preclude a potential therapeutic benefit from menin inhibition. For instance, despite normal expression of *HOXA* and *MEIS1,* perturbation of the menin-KMT2A complex impairs proliferation in leukemia cell lines of mutated CEBPα AML (Schmidt et al., 2019). Ongoing and future translational studies may resolve the role of the menin-KMT2A complex in leukemogenesis across various gene expression signatures and refine the identification of AML subgroups susceptible to menin inhibition.

Preliminary results from current clinical trials have provided evidence of manageable toxicity profiles and promising early efficacy signals of menin inhibition in heavily pretreated patients with *KMT2A*-r and NPM1c AML. Differentiation syndrome is an expected and potentially lethal toxicity but may be mitigated by early initiation of chemotherapy and combination regimens will be investigated in a number of clinical trials. At this moment, data from children treated with menin inhibitors is sparse but efforts to design pediatric trials and gain confidence in pediatric dosing, drug-drug and drug-food interactions and reproducible efficacy across genetic subtypes are ongoing (ACCELERATE virtual meeting on Menin inhibitors, 2022). Current salvage therapies of R/R pediatric AML are associated with significant treatment-related morbidity and mortality (Molgaard-Hansen et al., 2011) and further improvement of outcomes call for highly effective therapies without detrimental off-target toxicities to accompany or even replace current therapy elements. Consolidation by SCT may still imply the highest chance of permanent cure in future treatment of R/R AML, however, addition of targeted agents such as menin inhibitors may potentially allow mitigation of treatment intensity on other parameters, e.g. omission of anthracyclines and consequently reduction of short- and long-term cardiac toxicities (Barlogis et al., 2015; Jarfelt et al., 2016). Moreover, the favorable toxicity profile of this class of agents provides rationale to study menin inhibition as maintenance therapy in the post-transplant setting. This approach has demonstrated promising results in AML with other targeted agents such as tyrosine kinase inhibitors and demethylating agents (Bewersdorf et al., 2021).

The need for development of biomarker-driven treatment approaches in pediatric AML is indisputable. However, considering the number of commercially available menin inhibitors and potential combination regimens with both standard chemotherapy backbones and other targeted agents, coordination and prioritization of trials within a limited sub-population of children with AML raise significant challenges. The absolute number of children with relapsed AML suitable for investigational treatment with menin inhibitors does not exceed a total of 160 new cases per year in collaborative study groups from Europe and North America (Table 1). Furthermore, conductance of phase I/II clinical trials with agents targeting the *HOXA*/*MEIS1* transcription program is challenged by scarcity of comparison cohorts with annotated response data, geographical differences in regulatory filing and patient access. These challenges call for careful coordination of future trials, e.g. by collaborative efforts such as the ACCELERATE and PedAL/EUPAL initiatives, that aim to facilitate recruitment and allocation of children to the most appropriate evidence-based and, when feasible, biomarker-driven trial in order to maximize the chance of cure and the amount of informative research within a limited and heterogeneous patient population. Prioritization of infrastructure for efficient development and implementation of pediatric trials with new, promising agents like menin inhibitors is critical to ensure therapeutic progress in childhood AML.

## Figures and Tables

**Table 1:** Clinical characteristics of genetic entities in pediatric AML characterized by aberrant *HOXA*/*MEIS1* expression.

Genetic subgroup	Frequency	Cases per year (newly diagnosed/relapse)^a^	Age at diagnosis (median)	Risk group	Outcome (mean)^b^
***KMT2A*-rearrangements** Subgroup by fusion partner:	20–25%	200/100	2 years		EFS: 40–45% OS: 55–60%
• Xq24/*SEPT6*, 1q21/*MLLT11*, 9p22/*MLLT3*, 17q21/*MLLT6*, 19p13.1/ELL	10%	90/30		Intermediate	EFS: 40–90% OS: 65–95%
• 4q21/*AFF1*, 6q27/*MLLT4*, 10p11.2/*ABI1*, 10p12/*MLLT10*, 19p13.3/*MLLT1*	7%	60/45		Adverse	EFS: 10–40% OS: 20–50%
• Other or unknown	5%	45/20		Indeterminate	EFS: 40–45% OS: 50–55
Therapy-related *KMT2A*-r AML	<1%	5/3	+3 years^c^	Adverse	OS: <30%
***NPM1* mutation**	8%	65/10	11 years	Favorable	EFS: 65% OS: 70–80%
**NUP98-rearrangements** Subgroup by fusion partner:	5–10%	60/45	10 years		
• 5q35/*NSD1*	4%	30/25	13 years	Adverse	EFS 10–15% OS: 30–50
• 12p13/*KDM5A*	2%	15/10	3 years	Adverse	EFS: 30% OS: 35%
• Other or unknown	2%	15/10	variable	Adverse	NA

Table content is based on results from published cohort studies in *KMT2A*-r (Balgobind et al., 2009; Pollard et al., 2021; Weelderen et al., 2023; Schwartz et al., 2021), *NPM1* mutated (Brown et al., 2007; Hollink et al., 2009; Xu et al., 2020) and *NUP98*-r AML (Hollink et al., 2011; Bisio et al., 2017; Niktoreh et al., 2019; Struski et al., 2017).

a Estimated numbers in Europe and North America.

b Outcome is denoted as rounded mean event-free and overall survival estimates (EFS and OS) at 5 years (*KMT2A*-rearrangements, *NPM1* mutations, *NUP98*::*KDM5A* fusions) and 3 or 4 years from diagnosis (*NUP98::NSD1* fusions).

c Median time from primary cancer diagnosis (range: 1–15 years) (Schwartz et al., 2021).

**Table 2:** Schematic overview of menin inhibitors in clinical trials currently recruiting or in development for patients with AML.

Company	Drug	Trial (ClinicalTrials.gov ID)	Phase	Regimen	Patient population	Key eligibility	Status
Syndax Pharmaceuticals	Revumenib	AUGMENT-101 (NCT04065399)	I/II	Revumenib monotherapy	Adults and children	R/R *KMT2A*-r AL, NPM1c AML	Recruiting
		AUGMENT-102 (NCT05326516)	I	AML: revumenib/FLA ±revumenib/FLA ALL/MPAL: revumenib/Pred/VCR/ASP/DNR±ETO/CPM	Adults and children	R/R *KMT2A*-r AL, NPM1c or *NUP98*-r AML	Recruiting
		SAVE(NCT05360160)	I/II	Revumenib/ASTX727/VEN	Adults and children	R/R AML or MPAL	Recruiting
		BeatAML substudy NCT03013998)	I	Revumenib/VEN/AZA	Adults	Newly diagnosed *KMT2A*-r or NPM1c AML	Recruiting
Kura Oncology	Ziftomenib	Komet-001 (NCT04067336)	I/II	Ziftomenib monotherapy	Adults	Phase Ia: R/R AML Phase Ib/II: R/R *KMT2A*-r or NPM1c AML	Recruiting
		Komet-007 (NCT05735184)	I	Newly diagnosed AML: ziftomenib/7 + 3 R/R AML: ziftomenib/VEN/AZA	Adults	Newly diagnosed and R/R *KMT2A*-r or NPM1c AML	Not yet recruiting
Janssen	JNJ-75276617	NCT04811560	I	JNJ-75276617 monotherapy	Adults	R/R *KMT2A*-r AL, NPM1c AML	Recruiting
		NCT05453903	I	JNJ-75276617/VEN, JNJ-75276617/AZA or JNJ-75276617/VEN/AZA	Adults	R/R *KMT2A*-r or NPM1c AML	Recruiting
		NCT05521087	I	AML: JNJ-75276617/FLA ALL: JNJ-75276617/DEX/VCR/ASP	Adults and children	R/R *KMT2A*-r ALL/AML, NPM1c or *NUP98*-r AML	Not yet recruiting
Biomea Fusion	BMF-219	COVALENT-101 (NCT05153330)	I	BMF-219 monotherapy	Adults	R/R AL, DLBCL or MM	Recruiting
Daiichi Sankyo	DS-1594	NCT04752163	I/II	DS-1594 ± AZA, VEN, or mini-HCVD	Adults	Phase I: R/R AL Phase II: R/R *KMT2A*-r ALL/AML, NPM1c AML	Active, not recruiting
Sumitomo Dainippon Pharma	DSP-5336	NCT04988555	I/II	DSP-5336 monotherapy	Adults	Phase I: R/R AL Phase II: R/R *KMT2A*-r ALL/AML, NPM1c AML	Recruiting

Trial status retrieved March 15, 2023 from clinicaltrials.gov.

AL, acute leukemia; ALL, acute lymphoblastic leukemia; AML, acute myeloid leukemia; ASP, PEG-asparaginase; AZA, azacytidine; CPM, cyclophosphamide; DEX, dexamethasone; DNR, daunorubicine; DLBCL, diffuse large B-cell lymphoma; ETO, etoposide; FLA, fludarabine and cytarabine; MPAL, mixed phenotype acute leukemia; mini-HCVD, cyclophosphamide, cytarabine, dexamethasone, methotrexate, prednisolone, rituximab and vincristine; MM, multiple myeloma; Pred, prednisolone; R/R, refractory or relapse; VCR, vincristine; VEN, venetoclax; 7 + 3: cytarabine and anthracycline.

## Data Availability

No data was used for the research described in the article.
